# Assessing the Impact of Maitland Mobilization Combined With a Conventional Physiotherapy Regimen Using the Visual Analog Scale and Disability of the Arm, Shoulder and Hand Scale in Diabetic Frozen Shoulder With Moderate Tissue Irritability Level

**DOI:** 10.7759/cureus.74640

**Published:** 2024-11-27

**Authors:** Vaishali Jagtap, G Varadharajulu

**Affiliations:** 1 Orthopaedics, Krishna College of Physiotherapy, Krishna Vishwa Vidyapeeth, Karad, IND; 2 Physical Medicine and Rehabilitation, Krishna College of Physiotherapy, Krishna Vishwa Vidyapeeth, Karad, IND

**Keywords:** diabetic, frozen shoulder, maitland mobilization, moderate tissue irritability, physiotherapy

## Abstract

Background

Patients suffering from diabetic frozen shoulder face particular challenges, including limited shoulder motion and excruciating pain. Although traditional physiotherapy methods are commonly used, it is important to investigate the effectiveness of combining Maitland mobilization with traditional treatments, especially considering patients’ tissue irritability levels as often patients cannot tolerate mobilization and exercises due to tissue irritability. This study aimed to investigate the impact of Maitland mobilization combined with a conventional physiotherapy regimen on pain and functional ability using the Visual Analog Scale (VAS) and Disability of the Arm, Shoulder and Hand (DASH) scales in diabetic patients suffering from a frozen shoulder with moderate tissue irritability levels.

Methodology

A total of 30 diabetic patients suffering from frozen shoulders with moderate irritability levels were assigned to two groups. Group A was administered conventional physiotherapy which consisted of hot moist packs, therapeutic ultrasounds, and exercises. Group B received conventional therapy with Maitland mobilization. The treatment lasted for six weeks with four sessions per week. Pre- and post-treatment pain and disability were assessed using the VAS and DASH scales.

Results

Post-treatment Groups A and B showed significant improvements. In Group A, the mean VAS score reduced from 4.97 ± 0.26 to 4.00 ± 0.19 (p < 0.001), while Group B showed a greater reduction from 5.00 ± 0.21 to 2.71 ± 0.13 (p < 0.001). Both groups showed an improvement in DASH scores, with the DASH score decreasing from 27.17 ± 2.37 to 21.89 ± 1.89 in Group A (p < 0.001). Group B showed more significant improvement from 27.23 ± 1.73 to 15.12 ± 0.78 (p < 0.001). Intergroup analysis between both groups showed significantly better outcomes in Group B in both VAS and DASH scores (p < 0.001).

Conclusions

Maitland mobilization with conventional physiotherapy significantly decreases pain and improves functional recovery in diabetic patients suffering from frozen shoulders with moderate tissue irritability levels.

## Introduction

Frozen shoulder, also termed adhesive capsulitis, is a musculoskeletal condition where patients experience pain and significant limitations in both active and passive shoulder range of motion [1]. Overall, 2-5% of the general population is affected by frozen shoulder, with a 10-20% higher prevalence in diabetes mellitus cases [2,3]. Diabetic patients suffering from frozen shoulders often have severe symptoms and experience a prolonged recovery period [4,5].

Pathophysiology of diabetic frozen shoulder involves inflammatory processes that lead to capsular fibrosis and contracture, which is exacerbated by altered glucose metabolism and the accumulation of advanced glycation end-products (AGEs) which is characteristic of diabetes [6,7]. Adhesive capsulitis in diabetic patients significantly affects patients’ quality of life, often resulting in pain and substantial disability, as measured by the Disability of the Arm, Shoulder and Hand Scale (DASH) scale [8,9].

Physiotherapy plays a central role in the primary treatment approach in disease management [10,11]. Conventional physiotherapy protocols typically consist of thermal modalities, therapeutic ultrasounds, and exercises [12,13]. However, in recent years, the addition of manual therapy techniques, particularly Maitland mobilization, has gained increased attention [14].

Maitland mobilization operates on the principle of oscillatory movements which are applied at varying grades to improve joint mobility and modulate pain [15,16]. The effect of the Maitland mobilization technique is attributed to its neurophysiological effects on mechanoreceptors and its ability in a controlled manner to stretch the capsular tissue [17]. Grade II mobilization when specifically applied to a frozen shoulder is relevant for moderate tissue irritability, as it works within the pain-free range while promoting mechanical effects on the capsular tissue [18].

Conventional physiotherapy along with Maitland mobilization may be beneficial, especially in managing moderate tissue irritability levels [19]. This combined approach using conventional physiotherapy along with Maitland mobilization addresses both the neurophysiological and mechanical aspects of this painful condition leading to improved pain and functional recovery in adhesive capsulitis [20,21].

Despite the theoretical basis supporting using conventional physiotherapy along with Maitland mobilization, there remains a need for clinical evidence, specifically examining its effectiveness in diabetic patients suffering from frozen shoulders. This study aims to examine the impact of Maitland mobilization combined with conventional physiotherapy regimens on pain and disability using the Visual Analog Scale (VAS) and DASH in diabetic patients suffering from frozen shoulders with moderate tissue irritability levels.

## Materials and methods

This study was approved by the Institutional Ethics Committee of Krishna Vishwa Vidyapeeth, Karad (approval number: KIMSDU/IEC/06/2022). A total of 30 diabetic patients diagnosed with moderate irritability frozen shoulder were assigned to the following two groups: Group A (conventional therapy) and Group B (Maitland mobilization + conventional therapy). Inclusion criteria included pain severity (VAS 4-6), moderate functional disability (DASH), and restricted active and passive range of motion with pain at the extreme range. Exclusion criteria included any shoulder surgery or trauma and high and low tissue irritability levels.

Patients in the conventional treatment Group A received 10 minutes of hot moist packs and continuous therapeutic ultrasound at 1.5 W/cm² for 10 minutes, followed by exercises such as wand exercises, pulley exercises, finger wall ladder, and capsular stretching. Patients in Group B received Maitland Grade II joint mobilizations (three sets) at the affected shoulder joint combined with the conventional protocol.

Before patients were included in the study, their informed consent was obtained, following which they were divided into two groups. Group A received conventional physiotherapy plus the Maitland mobilization approach, while Group B received conventional physiotherapy four days a week for six weeks. The VAS and DASH scores were used as outcomes before and after the treatment.

The data were analyzed statistically using paired t-tests to compare pre- and post-treatment values within each group and independent t-tests to compare outcomes between groups. The threshold for statistical significance was p-values <0.05. The mean of VAS, DASH, and standard deviation (SD) for VAS and DASH scores were calculated for both pre- and post-intervention periods.

## Results

Both groups showed notable improvements in pain and DASH scores after the intervention. However, Group B (Maitland + conventional therapy) demonstrated greater reductions in both VAS and DASH scores compared to Group A (conventional therapy), with significant differences between the groups post-intervention (Tables 1-3).

**Table 1:** Pain scores (VAS) for Group A and Group B. VAS: Visual Analog Scale

Group	Pre-VAS (mean ± SD)	Post-VAS (mean ± SD)	t-value	P-value
Group A (conventional therapy)	4.97 ± 0.26	4.00 ± 0.19	13.84	<0.001
Group B (Maitland + conventional therapy)	5.00 ± 0.21	2.71 ± 0.13	43.75	<0.001

**Table 2:** DASH Scores for Group A and Group B. DASH: Disability of the Arm, Shoulder and Hand

Group	Pre-DASH (mean ± SD)	Post-DASH (mean ± SD)	t-value	P-value
Group A (conventional therapy)	27.17 ± 2.37	21.89 ± 1.89	10.30	<0.001
Group B (Maitland + conventional therapy)	27.23 ± 1.73	15.12 ± 0.78	35.04	<0.001

**Table 3:** Statistical comparison between groups (post-intervention). DASH: Disability of the Arm, Shoulder and Hand; VAS: Visual Analog Scale

Variable	Group A (mean ± SD)	Group B (mean ± SD)	t-value	P-value
Post-VAS	4.00 ± 0.19	2.71 ± 0.13	21.24	<0.001
Post-DASH	21.89 ± 1.89	15.12 ± 0.78	17.48	<0.001

## Discussion

This study aimed to determine the impact of Maitland mobilization combined with a conventional physiotherapy regimen on pain and functional disability using VAS and DASH scores in diabetic patients suffering from frozen shoulders with moderate tissue irritability levels. The outcome measures used were pain relief and disability assessment using VAS and DASH, respectively.

The study findings demonstrated that both interventions, namely, conventional therapy alone (Group A) and Maitland mobilization plus conventional therapy (Group B), led to significant reductions in pain and improvements in disability. However, Group B showed a more significant improvement in both parameters, showing that Maitland mobilization enhances the significant improvement seen with the conventional rehabilitation protocol.

Both groups demonstrated a statistically significant decrease in VAS post-treatment. Group A, which received only conventional therapy, showed a mean VAS reduction from 4.97 to 4.00 (p < 0.001), while Group B, which received Maitland mobilization combined with conventional therapy, had a mean VAS reduction from 5.00 to 2.71 (p < 0.001). The greater reduction in VAS scores in Group B suggests that Maitland mobilization is more effective in relieving pain than conventional therapy alone. The significant reduction in VAS scores in Group B aligns with the findings of Kumar et al. [15] which demonstrated that Maitland mobilization effectively reduces pain in frozen shoulder.

Maitland Grade II mobilization involves passive oscillatory movements applied to the affected shoulder joint to decrease pain without increasing irritability. This technique likely reduces mechanical stress on the joint capsule and surrounding tissues, which is a main cause of pain in frozen shoulders, particularly in diabetic patients with extreme sensitivity to pain due to the glycosylation of collagen fibers. Moreover, the reduction in pain could also be attributed to the neuromodulatory effects of joint mobilization, which may decrease nociceptive input from the joint and associated structures. The neurophysiological pain reduction mechanism can be attributed to mechanoreceptor stimulation, as explained by Vicenzino et al. [16]. The enhanced pain reduction in diabetic patients can be understood through Zreik et al.’s research [2] on the connection between diabetes and adhesive capsulitis.

Regarding functional disability, both groups showed significant improvements in DASH scores. Group A mean DASH score varied from 27.17 to 21.89 (p < 0.001), while Group B demonstrated a more significant improvement, with the mean DASH score decreasing from 27.23 to 15.12 (p < 0.001). The improved functional recovery in Group B indicates that Maitland mobilization has a positive effect not only on pain reduction but also on the restoration of shoulder function.

Frozen shoulder symptoms include pain and stiffness, leading to decreased active and passive range of motion, significantly affecting daily activities. Conventional therapy, which consists of heat therapy and therapeutic ultrasound followed by active exercises such as wand exercises and capsular stretching, is designed to improve tissue extensibility and promote mobility. However, Maitland mobilization likely enhanced these effects by targeting joint mechanics more directly, allowing for improved joint play, better movement patterns, and greater range of motion during daily activities. The superior DASH score improvements in Group B support the findings of Vermeulen et al. [13] regarding the effectiveness of mobilization techniques. The improvement in functional outcomes aligns with Yang et al.’s research [18] on the effectiveness of end-range mobilization.

Clinical implications

The study findings support Page et al.’s Cochrane review [9] which emphasized the importance of manual therapy for frozen shoulder management. The specific benefits for diabetic patients align with Shah et al.’s research [4] on upper extremity impairments in diabetes.

Pathophysiological basis

The improvement mechanism can be understood through Bunker and Anthony’s work [5] on the pathology of frozen shoulders, especially with regard to tissue extensibility and joint mechanics. The treatment outcomes support Jain and Sharma’s systematic review [19] on physiotherapeutic interventions in frozen shoulders.

Treatment protocol effectiveness

The success of combining conventional therapy with Maitland mobilization supports Noten et al.’s systematic review findings [20] on the efficacy of mobilization techniques. The clinical approach aligns with the guidelines by Kelley et al. [10] for impairments in mobility and shoulder pain in adhesive capsulitis. The study on the effectiveness of Grade II mobilization aligns with the study by Johnson et al. [17] on joint mobilization in adhesive capsulitis.

Limitations

The lack of a control group and the need for further studies with longer follow-ups and larger sample sizes are the limitations of this study.

## Conclusions

Maitland mobilization, when combined with a conventional physiotherapy protocol, is more successful than conventional therapy alone in lowering pain and enhancing functional outcomes in diabetic patients with moderate irritability frozen shoulder. Physiotherapists should consider incorporating joint mobilization techniques to optimize recovery in this patient population.
